# Quality Evaluation of Small Features Fabricated by Fused Filament Fabrication Method

**DOI:** 10.3390/ma18030507

**Published:** 2025-01-23

**Authors:** Dawid Zieliński, Mariusz Deja, Rui Zhu

**Affiliations:** Department of Manufacturing and Production Engineering, Faculty of Mechanical Engineering and Ship Technology, Institute of Machine and Materials Technology, Gdańsk University of Technology, G. Narutowicza Str. 11/12, 80-233 Gdańsk, Poland; mariusz.deja@pg.edu.pl (M.D.); ruiz0925@163.com (R.Z.)

**Keywords:** additive manufacturing, fused filament fabrication method, quality control method, small-diameter features

## Abstract

The purpose of this research was to evaluate the quality of small features fabricated by the fused filament fabrication (FFF) method. The samples containing circular and square cross-sections through holes with different dimensions, lengths, and orientation angles were printed from ABS (acrylonitrile butadiene styrene) filament. The adopted optical inspection method allowed us to conduct observations of individual features and their measurements. The image processing software was used to determine the accuracy of the dimensions and shape of different cross-sections. Feret’s diameters were used for the evaluation of shape accuracy by comparing them with theoretical dimensions assumed in a 3D CAD model. Considering the relationship between the real and theoretical dimensions of different features, general empirical equations for predicting the equivalent dimensions were developed. The proposed method of the quality evaluation of small features can be easily implemented and widely applied to other features, especially internal holes with different cross-sections made using various additive manufacturing methods.

## 1. Introduction

Micro- and nano-manufacturing technologies have been gaining more and more importance in different industrial sectors. The possibility of manufacturing precision parts with small shapes—e.g., applied in electronic devices—requires the use of appropriate processes as well as methods for assessing the quality of the components obtained. Three-dimensional printing technology offers new possibilities in the production of components with complex external and internal structures, including small-sized features [1,2]. Considering the many advantages of additive manufacturing, it seems that 3D-printing methods can play a crucial role in this process. As shown in [3,4], microscale AM related to the fabrication of functional micro-/nano-devices is one of the fastest growing areas of AM research.

Three-dimensional printing technology, also referred to as additive manufacturing (AM), is a set of methods where the fabricated component is created layer by layer based on a 3D CAD model. Three-dimensional printing methods can be classified into different groups, such as powder bed fusion, material and binder jetting, vat polymerization, as well as material extrusion [5]. In recent years, small-scale 3D printers for printing from plastics, referred as desktop and low-cost 3D printers, have become increasingly important in both the industrial and amateur settings. The relatively simple construction and operation of the devices, as well as the low costs of the process, mean that printed parts are increasingly used, among other things, in the fabrication of parts containing small-sized features.

Currently, filament-based methods such as FFF/FDM (fused filament fabrication/fused deposition modeling) are widely used for 3D printing. In this process, thermoplastic material in the form of a filament is extruded from printer nozzles and then distributed layer by layer on the base platform. The most commonly used materials are ABS (acrylonitrile butadiene styrene), PLA (polylactic acid), PEEK (polyether ether ketone), PCL (polycaprolactone), as well as PC polycarbonate (polycarbonate) [6,7]. FFF/FDM methods can be used both in the industrial and academic sectors, as well as in amateur (hobby) settings. This is due to the relatively low cost of printers and materials used, as with low-cost 3D printers, mostly free software (slicer), and its simplicity [8,9]. Nowadays, there are several points of dynamic development of this technology. In addition to virgin and commercially available filaments such as ABS or PLA, hybrid or reinforced filaments are becoming increasingly important. Many studies have explored the use of additional fillers in the form of nanoparticles, glass, or carbon fibers to improve mechanical, electrical, and thermal properties compared to basic filaments [10,11,12]. Another important aspect is the analysis of key parameters influencing the surface quality (SQ) and the dimensional accuracy (DA) of FFF-/FDM-printed components. For example, the authors of the paper [13] divided FFF process parameters into three groups, i.e., the control, the signal, and the noise. Vidakis et al. [14] analyzed the effect of six control material extrusion parameters, such as layer thickness, printing speed, nozzle and bed temperatures, deposition angle and infill density, on the surface roughness, porosity, and dimensional accuracy of PLA parts. Then, the abovementioned parameters were used to develop the predictive regression models of the analyzed features. Newly introduced digital twins may overcome some challenges of AM by understanding the impact of processing parameters on the overall quality of the fabricated parts. By providing feedback information, real-time corrections can be performed [15]. Another approach is an extension of conventional 3D-printing technology based on Cartesian coordinates to a multi-axis system. Multi-axis additive manufacturing using printheads enables the production of parts with much more complex geometries and better surface quality compared to a three-axis process [16,17]. In general, the combination of subtractive and additive manufacturing methods defined as hybrid manufacturing as well as 4D printing are the key fabrication processes of Industry 4.0, which require the use of appropriate methods to evaluate the quality of generated parts [18].

Additive manufacturing is increasingly being used to build mechanical parts containing internal geometric features. Currently, powder bed fusion methods are widely used in the fabrication of such elements in the form of channels and cooling holes. In the paper [2], the DMLS (direct metal laser sintering) method was applied to build a model of a gas turbine blade with a designed series of holes and cooling channels with small diameters. In the next study, [19], authors used the SLS (selective laser sintering) method to fabricate a prototype abrasive tool also containing cooling holes with small diameters. The consideration of additional cooling allowed for a more efficient grinding process due to the reduced temperature in the cutting zone. Technologies based on material extrusion can also be successfully adopted to obtain this type of feature. At present, FDM systems are able to fabricate high-resolution microfluidic devices and microchannels [20,21]. For example, the authors of the paper [20] used low-cost FFF 3D printers to produce cooling channels with different diameters and shapes, such as linear, curved, spiral, and helical microchannels. However, microscopic observations indicated a failure to achieve the assumed dimensional and shape accuracy for the analyzed geometries, which significantly affects the performance of microfluidic devices. In [22], the authors proposed a geometrical model of the filament considering deposition angle and layer thickness to predict the dimensional accuracy of the FDM-printed part. For objects that improve accuracy in FFF/FDM, especially objects with small dimensions, it is necessary to develop appropriate measurement methods as well as design rules, i.e., design for additive manufacturing (DfAM) [23,24]. In industrial practice, one of the most popular methods for the inspection of features such as holes is the use of GO and NOT GO fixed gages [25]. Table 1 provides a comparison of contact and non-contact methods considering different criteria. Advanced measuring contact techniques, such as coordinate measuring machines, enable the performance of accurate and repeatable measurements. However, the irregular and rough surfaces of printed parts significantly limit the use of touch probes [26]. For internal geometrical features of manufactured parts, e.g., from metal powders and their alloys, it is possible to use non-contact methods based on digital radiography and computed tomography, which are relatively expensive. In the paper [1], authors demonstrated a fast and cheap method for evaluating dimensional and shape accuracy based on microscopic observations for samples made from metal powders by DMLS technology. The observed material defects in the form of burrs and tears resulted in smaller real hole diameters compared to their 3D CAD models.

The main objective of this study was to evaluate the dimensional and shape accuracy of small circular and square holes fabricated by the FFF method using acrylonitrile butadiene styrene ABS filament. For this purpose, the authors of this paper proposed a simple and inexpensive method of quality evaluation of small-diameter features based on optical inspection. A low-cost desktop FFF 3D printer allowed for the fabrication of samples containing circular and square cross-sections through holes from ABS filament. Moreover, the influence of dimensions, lengths, and printing orientations on their accuracy were investigated. As a result of the analysis performed, the ranges of achievable dimensions fabricated by the fused filament fabrication method were defined, and general empirical equations were developed to predict the dimensions of the circular and square cross-sections through holes.

## 2. Materials and Methods

### 2.1. Fabrication Process of the Test Samples

**Figure 1:**
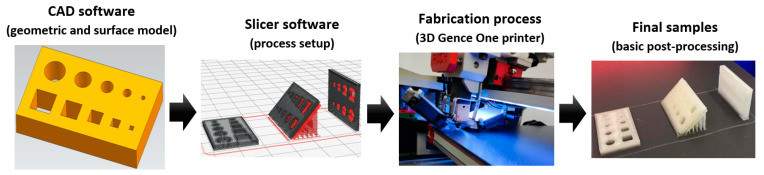
The process chain of the samples printed by the FFF method.

**Figure 2:**
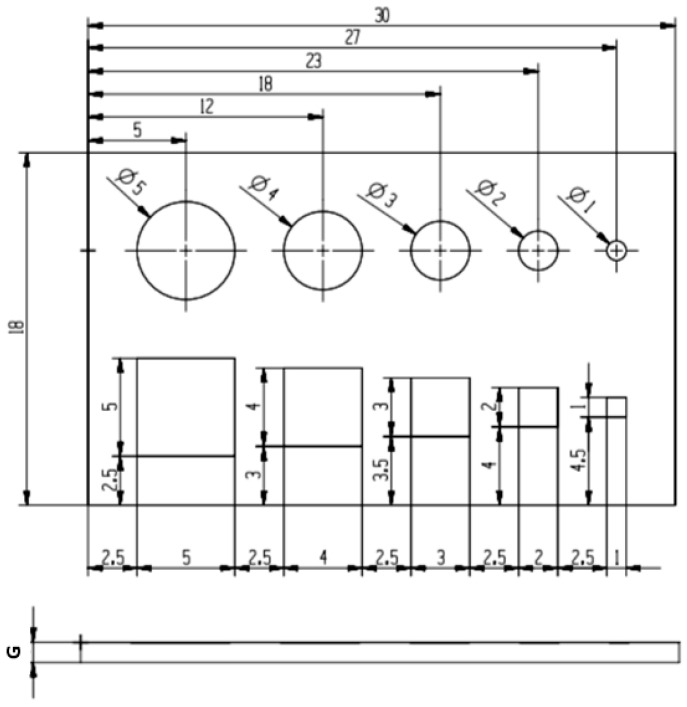
Technical drawing of test samples printed from ABS filament.

The fabrication process was performed on a commercially available 3DGence ONE printer (3DGence Sp. z.o.o., Przyszowice, Poland) with the following specifications: model: ONE; print technology: FFF (fused filament fabrication); number of printheads: 1; dedicated slicer software 3DGence Slicer using the process parameters given in Table 2. Test samples fabricated by the FFF method using ABS filament (3DGence Sp. z.o.o., Przyszowice, Poland)—whose specifications are given in Table 3, according to the process chain demonstrated in Figure 1—consisted of circular and square cross-sections through features of varying dimensions ranging from 1 to 5 mm (Figure 2). In samples of heights (*G)* 1 mm, 2 mm, and 3 mm, holes were designed and printed under 3 orientation angles: 0°, 45° and 90°. In the first step, samples containing small circular and square holes were designed by Autodesk Inventor Professional 2024 CAD software (San Francisco, CA, USA). After that, the designed models were saved as STL files to create surface geometries. 3DGence Slicer software (v4.0, 3DGence Sp. z.o.o., Przyszowice, Poland) was used to set all relevant slicing and process parameters, as given in Table 2. The set of process parameters was recommended by the slicing software according to the characteristics of the type of filament and the device used. In the final step, the models were cut into the specified layer thickness, while the generated program (G-code) was uploaded to the 3D printer. Support structures were generated automatically in the slicer software, considering the print orientation. After completing the building process, the samples were separated from the 3D-printer platform, and the support structures were removed (basic post-processing).

### 2.2. Research Methodology

**Figure 3:**
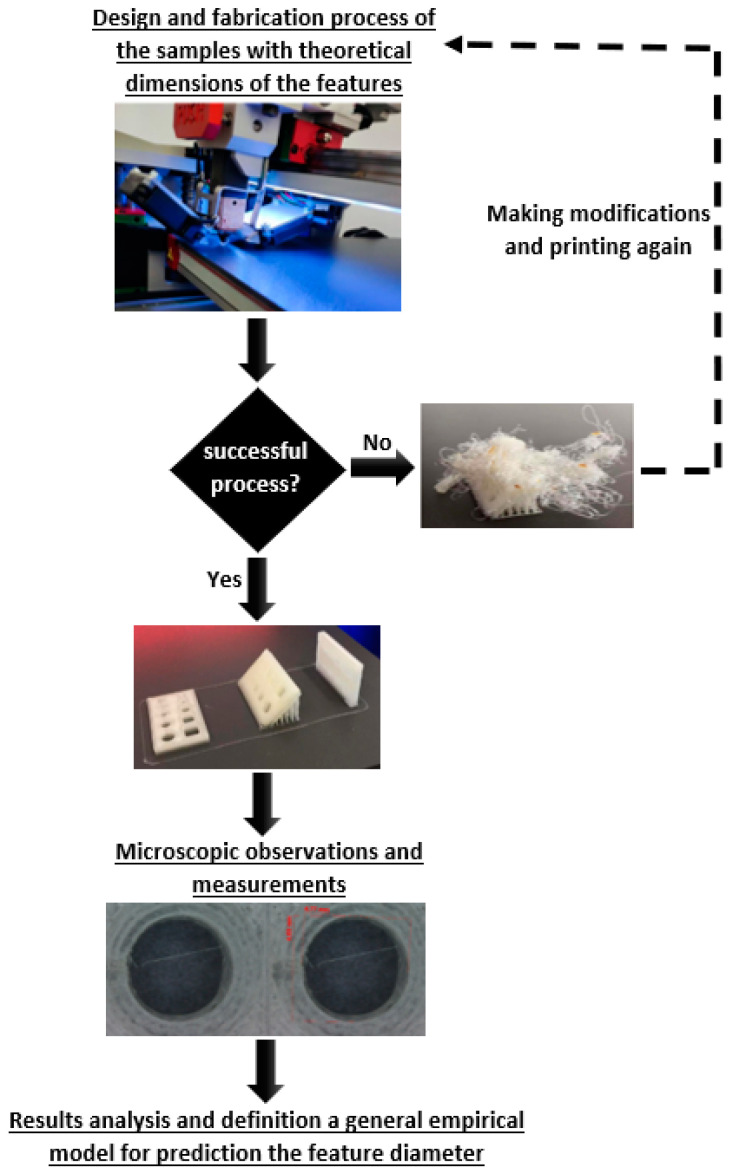
The adopted research methodology for evaluating the quality of small-diameter features fabricated by the FFF method.

The quality evaluation of small features fabricated by the fused filament fabrication method was carried out according to the procedure given in Figure 3. Firstly, after completing the printing process, the quality of the samples was assessed. In the case of unsuccessful printing of samples, the fabrication process was repeated. This was due to the anomalies that may occur during the fabrication, such as the element detaching from the platform during the printing process or the nozzle becoming clogged. When the process was successful, microscopic observations were made using a stereoscopic microscope Zeiss STEMI model 2000-C (ZEISS AG, Oberkochen, Germany) with total magnification levels of 6.5×–50× and dedicated AxioVision SE64 software v4.9.1. Considering the size of the samples and the analyzed features, the observations were performed at the lowest magnification, 6.5×, while the calibration was conducted using a standard gauge. The obtained series of images was used to perform measurements of the features in a graphical software.

## 3. Experimental Results

### 3.1. Quality Evaluation of Test Samples

**Figure 4:**
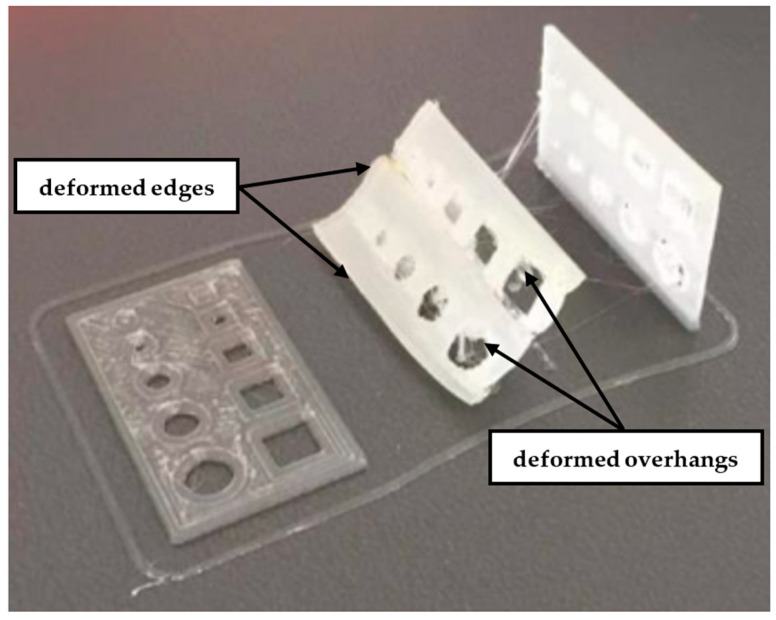
Test samples with a height of *G* = 1 mm after printing from ABS filament for three printing orientations—0°, 45°, and 90°—with examples of deformed features.

After finishing the printing process, the quality of test samples containing features of different shapes was assessed. Initial macroscopic observations indicated that most of them were successfully completed with the parameters used. Only in the case of samples printed at an angle of 45° were significant deformations observed, which made it impossible to obtain the full contours of the designed holes; see Figure 4. Despite repeating the process of printing them several times, a similar result was obtained each time, which was mainly due to the relatively low height *G* of the sample made of ABS material susceptible to deformation.

**Figure 5:**
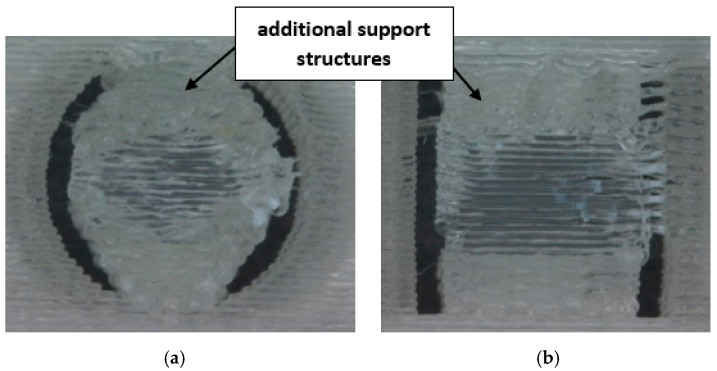
Examples of features produced for a 90° orientation with additional support structures: (**a**) circular and (**b**) square cross-sections.

Furthermore, for samples printed at a 90° orientation, it was necessary to use additional support structures for both the analyzed cross-sections; see Figure 5. Further analysis and measurements included only those holes that did not require the application of additional processing, in order to demonstrate the real possibilities of producing internal features with a specific clearance, without the use of post-processing.

### 3.2. Measurements and Quality Evaluation of Holes

**Figure 6:**
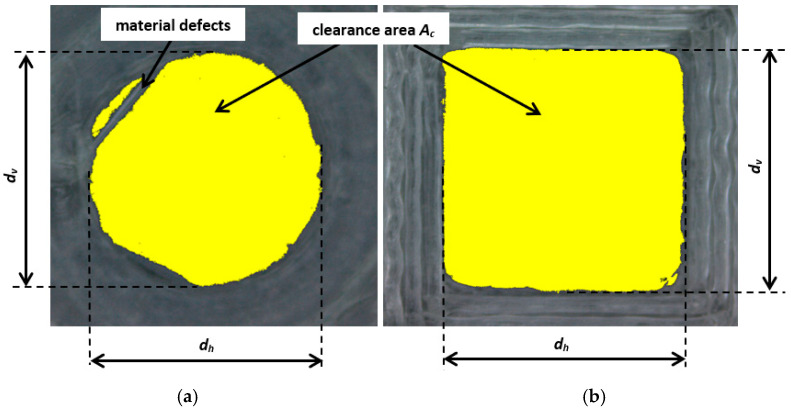
Top views of the features after the determination of their clearance and Feret’s diameters: (**a**) circular cross-section of an assumed diameter of *d_t_* = 4 mm, height of 1 mm, and printing orientation of 0°; (**b**) square cross-section of an assumed diameter of *d_t_* = 5 mm, height of 1 mm, and printing orientation of 0°.

The quality evaluation of the test samples after the printing process and a series of microscopic images allowed us to determine the achievable dimensional range of internal features. Software for image measuring and processing, MultiScan ver. 6.08 (Computer Scanning Systems, Warsaw, Poland), was used to determine the real clearance of the inner features. After thresholding, a yellow zone was assumed as the feature clearance (*A_c_*), and Feret’s diameters horizontal *d_h_* and vertical *d_v_* were assumed as measures of an object’s size along a specified direction; see Figure 6. Feret’s diameters were calculated automatically using software based on the threshold value adjusted for the best image quality. The equivalent feature diameter *d_e_* was calculated on a spreadsheet using the following formulas for circular cross-sections:

(1) $$A_{e}=A_{c}=\frac{\pi {d_{e}}^{2}}{4},$$

(2) $$d_{e}=2\sqrt{\frac{A_{c}}{\pi }},$$

and the following for square cross-sections:

(3) $$A_{e}=A_{c}={d_{e}}^{2},$$

(4) $$d_{e}=\sqrt{A_{c}},$$

where

*A_c_*—feature clearance;

*A_e_*—equivalent feature clearance;

*d_e_*—equivalent feature dimension.

As proposed in [1], the evaluation of the size and shape of fabricated features was based on the comparison of the calculated dimensions *d_e_*, *d_v_*, and *d_h_*, with the theoretical diameter *d_t_* assigned by the designer. Three conditions regarding the clearance and form errors can be determined for the quality evaluation:

*d_e_* = *d_h_* = *d_v_* = *d_t_* : full theoretical clearance, no shape error (5)

*d_e_* = *d_t_* ∧ (*d_e_* ≠ *d_v_* ∨ *d_e_* ≠ *d_h_*) : full theoretical clearance, shape error (6)

*d_e_* ≠ *d_t_* ∧ (*d_e_* ≠ *d_v_* ∨ *d_e_* ≠ *d_h_*) : clearance difference, shape error (7)

where

*d_t_*—theoretical diameter assigned in the CAD model;

*d_v_*—vertical Feret’s diameter;

*d_h_*—horizontal Feret’s diameter.

Finally, dimensions *d_e_*, *d_h_*, and *d_v_* were compared to a theoretical dimension *d_t_*, assigned in a CAD software, and the absolute Δ*d* and relative *δ_d_* errors were calculated according to the following equations:

Δ*d* = *d_t_* − *d_e_*, (8)

*δ_d_* = (Δ*d*/*d_t_*) · 100%. (9)

The calculated diameters and errors for the circular and square cross-sections are presented in Table 4 and Table 5, respectively.

**Figure 7:**
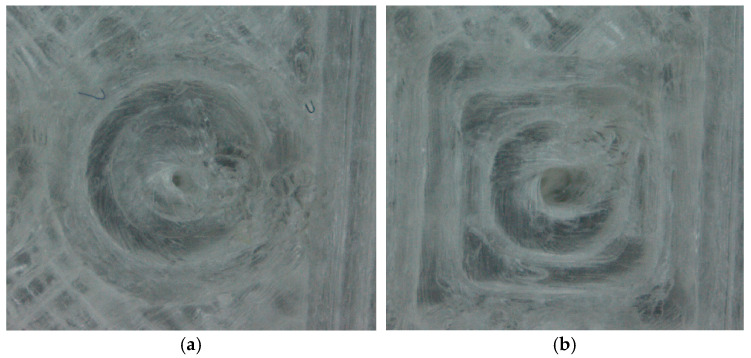
Examples of features with the assumed dimension *d_t_* = 1 mm without obtaining any clearance: (**a**) circular and (**b**) square cross-sections.

**Figure 8:**
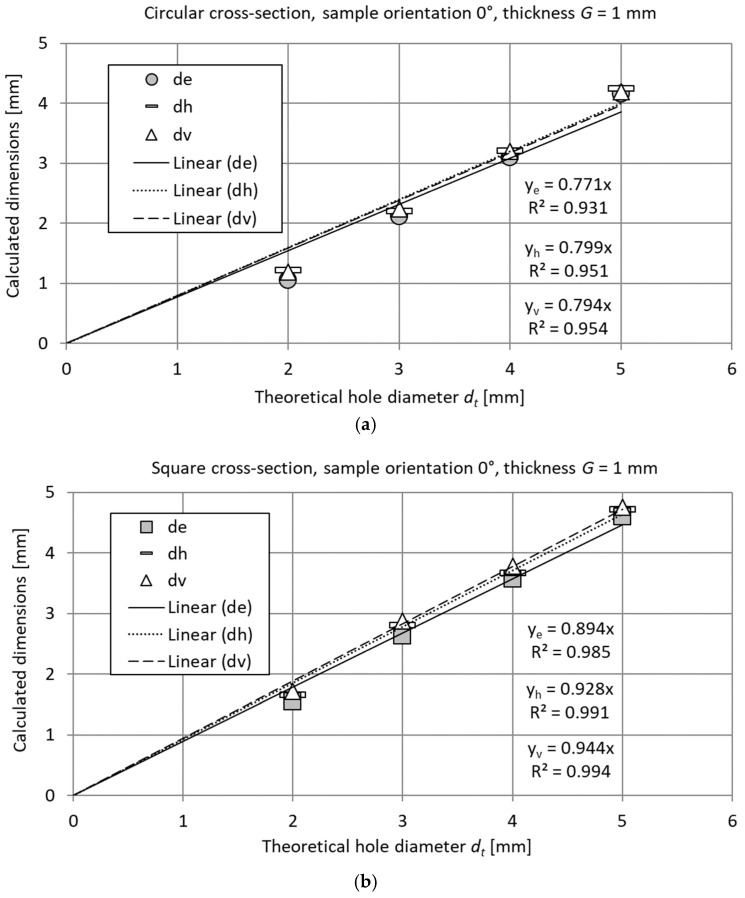
Feature dimensions *d_e_*, *d_v_*, and *d_h_* determined for exemplary test samples containing (**a**) circular and (**b**) square cross-section features for an orientation of 0° and a thickness of *G* = 1 mm.

An analysis of the microscopic images indicated that clearances were obtained for most of the holes, except for round and square holes with an assumed diameter of *d_t_* = 1 mm. In this case, only the beginnings of holes without full clearances were observed; see Figure 7. Moreover, the assumed theoretical dimension *d_t_* could not be achieved for any of the holes. The material defects visible on the microscopic images reduced the real size of the holes. Meanwhile, the differences occurring between the calculated and determined values of *d_e_* and Feret’s diameters *d_h_* and *d_v_* indicate that the assumed shapes of the features were not obtained; see Figure 8. For each feature, the third condition of the difference in clearance and shape error was met; see Equation (9). Even small deviations between these dimensions indicate shape errors, especially roundness errors of the circular cross-section features and errors of straightness and the parallelism of the edges of the produced square cross-section features. All three diameters should be equal within the assumed tolerances for ideal round or square contours. The calculated differences, as well as the visual assessments of the images of individual holes, indicated that the round and square shapes were not fully obtained for any of the features. As expected, the diameters *d_e_*, *d_v_*, and *d_h_* were all smaller than the theoretical diameter *d_t_* assigned in the CAD model, and *d_e_* was usually smaller than *d_v_* and *d_h_*.

**Figure 9:**
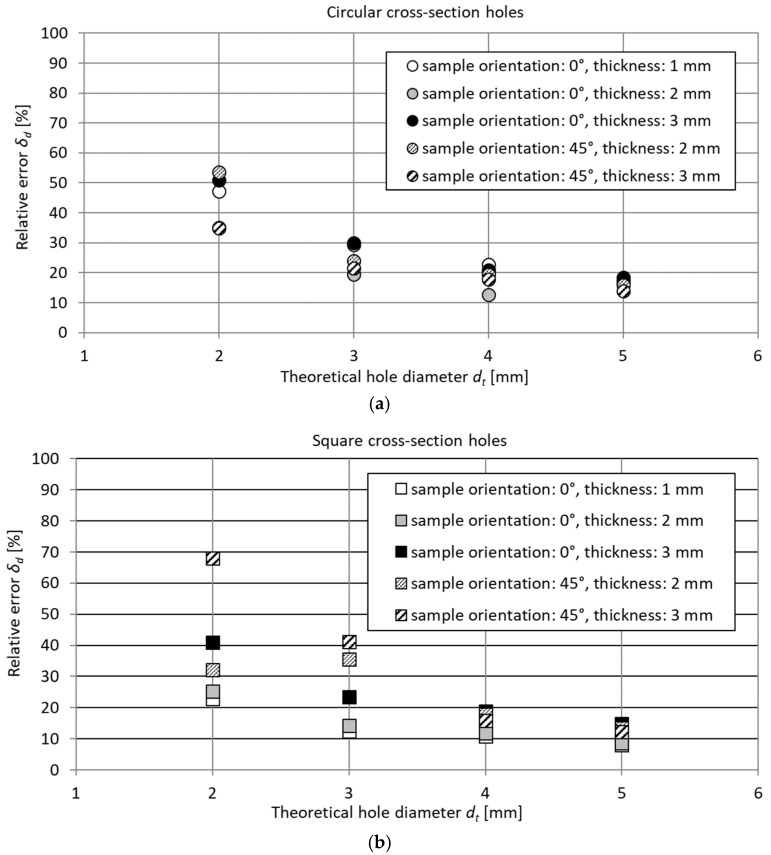
Relative errors of theoretical dimension *d_t_* for test samples containing circular (**a**) and square (**b**) cross-section features.

The differences between dimensions *d_t_* and *d_e_* were at a similar level for all dimensions and shapes, resulting in the value of relative errors decreasing with increasing dimensions; see Figure 9. A similar trend was observed for each of the analyzed printing orientations and feature lengths.

### 3.3. Regression Equations for Determining a Clearance of Circular and Square Cross-Section Features

Predicting dimensions is crucial for designing the features of mechanical components, especially when there is limited or no access to them during post-processing to improve their quality. By fitting mathematical equations to experimental data, a theoretical diameter *d_t_* can be better assigned to CAD models used to fabricate a feature with the required clearance. According to the trends of the obtained results shown in Figure 8, the dependencies between the calculated diameters *d_e_*, *d_v_*, and *d_h_* and the theoretical diameter *d_t_* were approximated by the following linear function:

(10) $$\hat{y}=a\;\cdot \;d_{t}$$

where

$\hat{y}—$the expected value of dimension *d_e_*, *d_v_*, or *d_h_*;

*a*—the directional coefficient of the regression function.

The fitting of the empirical data was evaluated using the coefficient of determination R^2^. The maximum absolute error Δ*y_max_* and the maximum relative error *δ*_max_ were calculated using the appropriate formulas:

(11) $$∆y_{max}=max\left|y_{i}-{\hat{y}}_{i}\right|,$$

(12) $$\delta_{\max}=\max\left(\frac{\left|y_{i}-{\hat{y}}_{i}\right|}{y_{i}}\right)100\%.$$

As seen in Table 6 and Table 7, the proposed function for calculating all the diameters enabled good fitting of the experimental data for samples of different thicknesses. The linear functions determined for square holes were characterized by the better fitting with the lower Δ*y_max_*, *δ*_max_, and RMSE errors, with the exception of diameter *d_e_* for a sample height of 3 mm and printing orientation 45°.

For the square holes and a printing orientation of 0°, the value of the directional coefficient *a* of a function for determining a hole’s diameter decreased with the increase in a sample’s thickness (Table 7). For the circular holes and a printing orientation of 0°, the value of the directional coefficient *a* remained stable with the increase in a sample’s thickness (Table 6). This allowed for the development of a general linear function for determining specific dimensions (hole clearances):

(13) $$\hat{a}=a_{d}G+b,$$

where

$\hat{a}—$the expected value of the directional coefficient of the linear regression function; 

*G*—the thickness of the sample;

*a_d_*, *b*—the coefficients of the function for determining specific dimensions for *d_e_*, *d_v_*, or *d_h_*.

**Figure 10:**
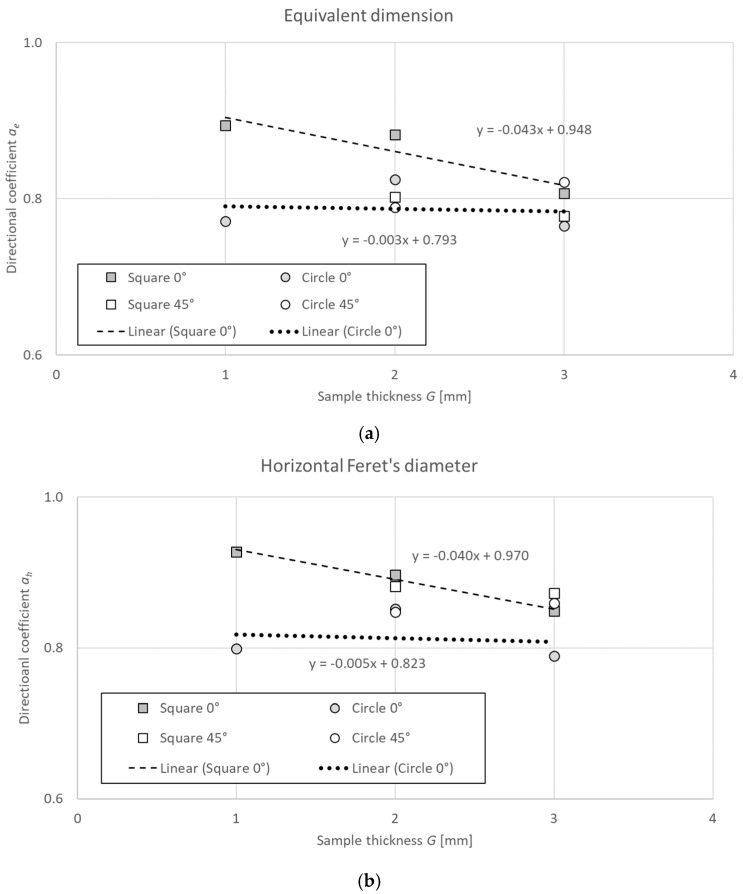

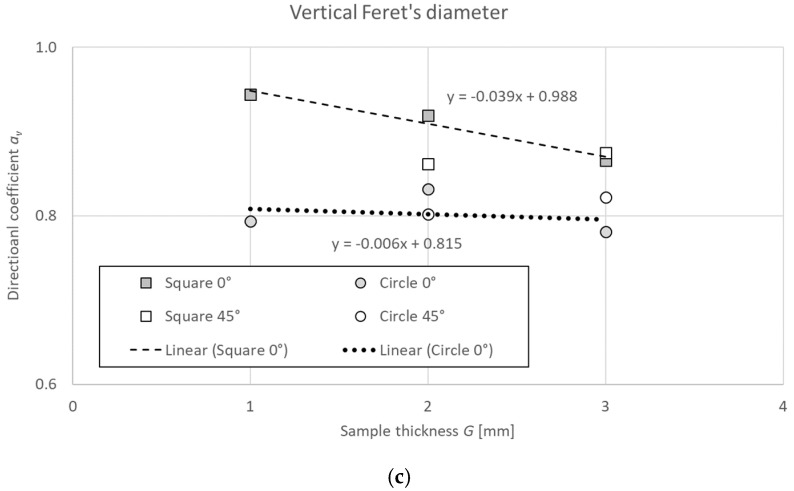
The relationship between hole length (sample thickness G) and directional coefficient a of a linear function determined for theoretical hole diameter d_t_ in the range of 2–5 mm: (**a**) equivalent dimension, (**b**) horizontal Feret’s diameter, and (**c**) vertical Feret’s diameter.

The assumed approximation function for the analyzed range of theoretical diameter *d_t_* = 2 ÷ 5 mm and that of sample thickness G = 2 ÷ 5 mm are presented in Figure 10.

## 4. Discussion

In this proposal, the authors demonstrate that a relatively simple and cost-effective optical inspection method has been successfully applied to assess the dimensional and shape accuracy of small features in the form of circular and square holes fabricated by the FFF method using virgin ABS filament. The study conducted indicated several limitations on the obtained dimensional and shape accuracies of the small features analyzed. Despite the adopted set of process parameters and the type of filament used, the proposed method allowed most of the designed features to be fabricated. The main limitations were the inability to achieve theoretical dimensions for the smallest features with an assumed diameter of *d_t_* = 1 mm as well as the requirement of generating support structures for holes printed at an angle of 90°. The abovementioned limitations resulted from several aspects related to the process parameters used, mainly layer thickness and printing speed, as well as the properties of the ABS feedstock and its behavior during the printing and cooling stages. In this case, it would be more appropriate to use a more advanced 3D printer in an enclosed workspace to maintain a constant temperature and controlled cooling of the filament, which is susceptible to deformation. In addition, changing the values of the process parameters could improve the accuracy of the features fabricated. In general, holes printed at an angle of 0° showed higher accuracy compared to holes fabricated at an angle of 45°. Subsequently, decreasing the dimensions of the holes and increasing their length may result in material defects that affect the accuracy of the printed features. Thus, the results of this study confirmed previous observations presented in [1,2,20] considering small-diameter features printed from metal powders and plastic feedstock when the appropriate measurements were conducted. Although coordinate measuring machines enable accurate inspection, they cannot be used to measure fabricated features characterized by small dimensions and irregular shapes, which limit access to the measuring zone. Non-contact techniques seem to be the most suitable for their assessment. Considering the cost and ease of use, the proposed methodology, based on optical inspection, allowed for the identification of defects and high measurement performance, as also shown in [20,21,29]. This indicates the universality of the proposed methodology for different AM methods. Therefore, the adopted optical inspection method can be successfully used for the systematic testing of features with different shapes and size, as well as features fabricated under different process parameters and 3D-printing methods.

The proposed optical inspection method aids in decision making for geometry acceptance and corrective actions in CAD software to meet design specifications. The developed mathematical functions assist designers in assigning diameters to ensure, e.g., proper fluid flow in channel holes. CAD software procedures and macros may support the hole design process for 3D-printing technologies.

## 5. Conclusions and Future Work

In this paper, we demonstrated the results of the quality evaluation of small-diameter holes created by low-cost fused filament fabrication and 3D-printed from ABS filament. The main conclusions are as follows:

- A general model for determining hole clearance was developed, allowing for the calculation of an equivalent hole diameter *d_e_* and Feret’s diameters *d_h_* and *d_v_* as a function of a sample thickness G = 2 ÷ 5 mm for the range of a theoretical diameter *d_t_* = 2 ÷ 5 mm.
- The adopted method of quality evaluation based on microscopic observations and image processing software is cheap, is easy to use, and can be applied to other 3D-printing technologies.
- Low-cost FFF 3D printer and the adopted set of RP process parameters made it possible to fabricate most of the designed round and square holes in the test samples, while failing to achieve the assumed dimensions and shapes.
- The differences between the diameters *d_e_*, *d_v_*, and *d_h_* indicate that the resulting internal profiles do not have fully circular and square shapes.
- Material defects have reduced the real dimensions of the holes compared to their CAD models, particularly for the smallest features.
- Printing holes at an angle of 90° require the use of support structures, while their manual removal may cause the deformation or damage of test samples with low heights. Therefore, it would be more beneficial to use soluble structures.

In future research, we will focus on a detailed statistical analysis of the results, which will allow us to evaluate the repeatability of the obtained dimensional and shape accuracies of the printed features. Thus, it will be necessary to fabricate more samples and use a wider range of RP process parameters. In particular, the influence of additional printing parameters—such as nozzle temperature, printing speed, as well as layer thickness—on the quality of the features obtained will be investigated. For example, based on our experience, using lower printing speeds and lower material thicknesses can improve the quality of the printed parts, including potentially small features, while increasing the 3D-printing process time. Therefore, further testing plans will consider different levels of variability in the analyzed parameters, which will simultaneously allow the development of more advanced and accurate mathematical models. Another important research direction is to conduct systematic testing not only for virgin filaments, such as ABS or PLA, but also for increasingly and widely used unconventional and hybrid materials.

## Figures and Tables

**Table 1:** Comparison of contact and non-contact methods for inspection of small features.

Comparative Criteria	Coordinate Measuring Machine	CT Scanning	Optical Inspection Method
Measurement type	Contact	Non-contact	Non-contact
Accuracy	Very high with repeatable measurements; possibility to compare the results with the CAD model	Very high and dependent on device type; possibility to compare the results with the CAD model	High and dependent on device type; no 3D model, only a 2D view
Cost	Very high, cost-effective solution for series production and measurement processes of complex shapes	Very high with the possibility of measuring complex shapes, including internal structures	High and dependent on device type, mainly measurements of outer surfaces
Efficiency	Very high with repeatable measurements; mainly dedicated for industry conditions	Lower and requiring the use of laboratory conditions	Lower and requiring the use of laboratory conditions
Type of material measured and measurement size	Limited due to the use of touch probes	Wide variety of material measured with the possibility of identifying internal material defects for the entire sample	Wide variety of material measured with the possibility of identifying material defects but only for dedicated surfaces
Measurement process	Complex, with various equipment required for measurement	Complex, with various equipment required for the measurement	Simple, with less equipment required for the measurement

**Table 2:** The parameters of the analyzed FFF process.

Parameter	Value
Material in filament form	ABS (3DGence)
Filament diameter [mm]	1.75
Nozzle diameter [mm]	0.4
Layer thickness [mm]	0.15
Print speed [mm/s]	50
Material temperature [°C]	250
Print bed temperature [°C]	105
Material of the print bed [-]	ceramic
Infill percent [%]	40
Infill pattern [-]	zig-zag
Bed adhesion [-]	skirt
Support material	same as model

**Table 3:** The specifications of the ABS material used, based on References [27,28].

General Information	
Company	3DGence
Batch number	M01119050701.00
Diameter [mm]	1.75
Color	white
Weight [kg]	1
Printed part density [kg/m^3^]	1040
**Mechanical properties**	
Tensile strength [MPa]	36.3 (XY) and 21.3 (ZX)
Flexural strength [MPa]	56.6 (XY), 58.3 (XZ), and 38.59 (ZX)
Flexural modulus [MPa]	1833 (XY), 1767 (XZ), and 1586 (ZX)
Young’s modulus [MPa]	1958 (XY) and 1608 (ZX)
Elongation at break [%]	7.4 (XY) and 1.8 (ZX)
Flexural strain at break [%]	5.3 (XY), 5 (XZ), and 3.1 (ZX)

**Table 4:** Measured dimensions and errors determined for test samples printed from ABS filament containing circular cross-section features.

Height of a Sample (Hole Length) [mm]	Printing Orientation	*d_t_* [mm]	*d_e_* [mm]	Δ*d* [mm]	*δ_d_* [%]	Feret’s Diameter	
						Horizontal *d_h_* [mm]	Vertical *d_v_* [mm]
1	0°	1	Not received				
		2	1.06	0.94	47.17	1.22	1.19
		3	2.12	0.88	29.36	2.20	2.24
		4	3.09	0.91	22.69	3.21	3.20
		5	4.16	0.84	16.74	4.25	4.19
2	0°	1	Not received				
		2	1.30	0.70	35.12	1.40	1.29
		3	2.42	0.58	19.42	2.54	2.45
		4	3.49	0.51	12.76	3.57	3.49
		5	4.14	0.86	17.11	4.26	4.20
	45°	1	Not received				
		2	0.93	1.07	53.57	1.51	1.00
		3	2.28	0.72	24.07	2.44	2.32
		4	3.22	0.78	19.48	3.46	3.24
		5	4.21	0.79	15.87	4.32	4.28
3	0°	1	Not received				
		2	0.98	1.02	51.02	0.99	1.09
		3	2.10	0.90	29.92	2.22	2.15
		4	3.16	0.84	20.96	3.25	3.23
		5	4.08	0.92	18.37	4.20	4.12
	45°	1	Not received				
		2	1.30	0.70	34.93	1.42	1.36
		3	2.35	0.65	21.55	2.48	2.32
		4	3.29	0.71	17.83	3.50	3.29
		5	4.31	0.69	13.82	4.42	4.31

**Table 5:** Measured dimensions and errors determined for test samples printed from ABS filament containing square cross-section features.

Height of a Sample (Hole Length) [mm]	Printing Orientation	*d_t_* [mm]	*d_e_* [mm]	Δ*d* [mm]	*δ_d_* [%]	Feret’s Diameter	
						Horizontal *d_h_* [mm]	Vertical *d_v_* [mm]
1	0°	1	Not received				
		2	1.54	0.46	22.76	1.66	1.72
		3	2.63	0.37	12.34	2.81	2.87
		4	3.57	0.43	10.77	3.68	3.78
		5	4.60	0.40	8.03	4.72	4.75
2	0°	1	Not received				
		2	1.49	0.51	25.34	1.66	1.67
		3	2.57	0.43	14.31	2.67	2.77
		4	3.53	0.47	11.86	3.54	3.68
		5	4.57	0.43	8.70	4.58	4.65
	45°	1	Not received				
		2	1.36	0.64	32.06	1.60	1.40
		3	1.94	1.06	35.44	2.57	2.45
		4	3.29	0.71	17.85	3.53	3.49
		5	4.33	0.67	13.42	4.51	4.48
3	0°	1	Not received				
		2	1.18	0.82	40.85	1.41	1.41
		3	2.30	0.70	23.45	2.40	2.52
		4	3.25	0.75	18.82	3.34	3.41
		5	4.26	0.74	14.75	4.49	4.54
	45°	1	Not received				
		2	0.64	1.36	67.92	1.47	1.51
		3	1.77	1.23	41.12	2.53	2.48
		4	3.37	0.63	15.79	3.52	3.56
		5	4.38	0.62	12.31	4.51	4.51

**Table 6:** Parameters of a function for calculating equivalent and Feret’s diameters for circular holes, *d_t_* = 2 ÷ 5 mm.

Sample Height (Hole Length) [mm]	Printing Orientation	Diameters [mm]	Directional Coefficient *a*	*R* ^2^	$∆y_{max}$ [mm]	*δ*_max_ [%]	*RMSE*
1	0°	*d_e_*	0.771	0.931	0.49	46.02	0.30
		*d_h_*	0.799	0.951	0.38	31.10	0.25
		*d_v_*	0.794	0.954	0.40	33.50	0.24
2	0°	*d_e_*	0.825	0.965	0.35	27.09	0.20
		*d_h_*	0.851	0.975	0.30	21.42	0.17
		*d_v_*	0.831	0.964	0.38	29.33	0.21
2	45°	*d_e_*	0.789	0.914	0.65	69.95	0.35
		*d_h_*	0.848	0.988	0.18	12.06	0.12
		*d_v_*	0.802	0.924	0.60	60.10	0.33
3	0°	*d_e_*	0.765	0.923	0.55	56.52	0.32
		*d_h_*	0.789	0.922	0.59	56.68	0.33
		*d_v_*	0.781	0.939	0.47	43.04	0.28
3	45°	*d_e_*	0.821	0.966	0.34	26.23	0.21
		*d_h_*	0.859	0.976	0.30	21.36	0.17
		*d_v_*	0.822	0.971	0.28	20.07	0.19

**Table 7:** Parameters of a function for calculating equivalent and Feret’s diameters for square holes, *d_t_* = 2 ÷ 5 mm.

Sample Height (Hole Length) [mm]	Printing Orientation	Diameters [mm]	Directional Coefficient *a*	*R* ^2^	$∆y_{max}$ [mm]	*δ*_max_ [%]	*RMSE*
1	0°	*d_e_*	0.894	0.985	0.24	15.68	0.14
		*d_h_*	0.928	0.991	0.19	11.57	0.11
		*d_v_*	0.944	0.994	0.17	9.98	0.09
2	0°	*d_e_*	0.882	0.980	0.27	18.13	0.16
		*d_h_*	0.897	0.994	0.13	7.82	0.08
		*d_v_*	0.919	0.994	0.16	9.86	0.09
2	45°	*d_e_*	0.802	0.928	0.47	24.26	0.31
		*d_h_*	0.882	0.991	0.16	10.00	0.10
		*d_v_*	0.861	0.971	0.32	22.83	0.20
3	0°	*d_e_*	0.807	0.951	0.43	36.37	0.25
		*d_h_*	0.849	0.967	0.29	20.37	0.21
		*d_v_*	0.866	0.970	0.32	22.87	0.20
3	45°	*d_e_*	0.777	0.823	0.91	142.33	0.61
		*d_h_*	0.873	0.979	0.28	18.80	0.16
		*d_v_*	0.875	0.980	0.24	15.94	0.16

## Data Availability

The original contributions presented in the study are included in the article, further inquiries can be directed to the corresponding authors.
